# Glutathione mitigates hypoxia-induced gill damage in juvenile grass carp (*Ctenopharyngodon idellus*) by alleviating endoplasmic reticulum stress and autophagy

**DOI:** 10.1186/s40104-025-01274-x

**Published:** 2025-11-06

**Authors:** Shenghua Zeng, Lin Feng, Pei Wu, Yang Liu, Yaobin Ma, Hongmei Ren, Xiaowan Jin, Xiaoqiu Zhou, Weidan Jiang

**Affiliations:** 1 Animal Nutrition Institute, Sichuan Agricultural University, Chengdu, Sichuan 611130 China; 2 Fish Nutrition and Safety Production University Key Laboratory of Sichuan Province, Sichuan Agricultural University, Chengdu, 611130 China; 3 Key Laboratory of Animal Disease-Resistance Nutrition, Ministry of Education, Ministry of Agriculture and Rural Affairs, Key Laboratory of Sichuan Province, Chengdu, 611130 China

**Keywords:** Apoptosis, Autophagy, Endoplasmic reticulum stress, Gills, Glutathione, Hypoxic stress

## Abstract

**Background:**

Environmental hypoxia is a common phenomenon in aquaculture, which causes gill tissue injury in fish. Glutathione (GSH) is a vital antioxidant in animal tissues, and its levels decrease under hypoxic conditions. However, the effects of glutathione on fish under hypoxic stress remain poorly understood. This study aimed to investigate the impact of glutathione on gill tissue damage in fish under hypoxic stress and explore the underlying mechanisms.

**Methods:**

Six experimental diets with varying glutathione concentrations. The actual glutathione levels in these diets, measured by high-performance liquid chromatography, were 0.00, 145.95, 291.90, 437.85, 583.80, and 729.75 mg/kg, respectively. Fish were fed these diets for 70 d, after which a 96-h hypoxic stress experiment was conducted. The experiment was set up with normoxic and hypoxic groups, in which the dissolved oxygen in the group was 6 mg/L, and that in the group was 1 mg/L.

**Results:**

This research revealed that glutathione could enhance the growth performance and antioxidant capability of juvenile grass carp while mitigating the structural damage to gill tissues induced by hypoxia stress. Mechanistic investigations further indicated that glutathione mitigated hypoxia-induced oxidative injury in gill tissues and improved their antioxidant capacity. In addition, glutathione attenuated gill apoptosis induced by hypoxia stress. Glutathione also inhibited the initiation, nucleation, elongation, and degradation phases of autophagy, thereby attenuating hypoxia-induced gill autophagy. Moreover, glutathione was found to alleviate hypoxia-induced endoplasmic reticulum stress (ERS) in gills, a response potentially linked to the suppression of PERK, IRE1, and ATF6 signaling pathways. Finally, based on the ROS and PC contents in gill tissue, the optimum glutathione supplementation levels for juvenile grass carp under hypoxia stress were 437.10 and 495.00 mg/kg, respectively.

**Conclusions:**

In conclusion, our experimental results demonstrated the effectiveness of glutathione in alleviating gill tissue damage caused by hypoxic stress. This study confirms the feasibility and effectiveness of dietary glutathione addition to alleviate hypoxic stress in fish.

**Supplementary Information:**

The online version contains supplementary material available at 10.1186/s40104-025-01274-x.

## Introduction

According to the State of World Fisheries and Aquaculture report released by the FAO in 2024, aquaculture accounted for 58.65 percent of global fisheries and aquaculture production in 2022 [1]. With the rapid development of the aquaculture industry, high-density and intensive farming methods often degrade water quality, exposing fish to various environmental stressors, inducing oxidative stress and growth retardation in fish [2]. In order to enhance the stress resistance of aquatic animals, exogenous antioxidant supplementation has been proven to be an effective strategy. Glutathione (GSH) is a vital non-enzymatic antioxidant in animal tissues. Our previous research revealed that glutathione effectively improved the growth performance of juvenile grass carp [3]. A previous study reported that glutathione enhanced the resistance of grass carp to microcystin-LR [4], suggesting that glutathione improved stress resistance in fish.

In addition, high-density intensive culture leads to a significant decrease in dissolved oxygen concentration in water, creating a hypoxic environment. Gills are important respiratory organs in fish, whose tissue structure and function are adversely affected by hypoxic stress. Moreover, hypoxic stress was found to lead to the proliferation and hypertrophy of gill epithelial cells in channel catfish [5], as well as the occurrence of gill filaments curl and engorgement in tilapia gill tissue [6]. Meanwhile, hypoxia results in excessive production of intracellular reactive oxygen species (ROS), which in turn cause oxidative injury in tissues [7], endoplasmic reticulum (ER) stress [8], autophagy and apoptosis [9]. A previous study has found that dietary glutathione supplementation could alleviate ammonia exposure-induced vascular congestion, cell vacuoles and inflammatory cell infiltration in carp gills [10]. However, no studies have explored the effect of glutathione on hypoxic stress-induced gill damage in fish, highlighting the relevance of such research.

Autophagy is a pathway that mediates the degradation of cytoplasmic proteins and organelles and is regulated by signaling molecules such as unc-51-like kinase 1 (Ulk1), coiled-coil myosin-like BCL2-interacting protein (Beclin1), microtubule-associated protein 1 Light chain 3 (Lc3) and autophagy receptor p62 (P62) [11]. Buthionine sulfoximine (BSO) treatment leads to depletion of intracellular glutathione levels and induces autophagy in spermatogonia and retinal pigment epithelial cells [12, 13]. Additionally, research in rats with hypoxic-ischemic brain injury has shown that glycine, a catabolic product of glutathione, could inhibit autophagy [14]. These studies imply that glutathione may play an inhibitory role in autophagy, and its specific mechanism needs further study.

Apoptosis is mediated by cysteine protease family caspases, which are divided into the endogenous apoptosis pathway and exogenous apoptosis pathway [15, 16]. When cells are stimulated by apoptotic signaling, Bcl2-associated X protein (Bax) induces the permeabilization of the outer mitochondrial membrane, activating the initiator Caspase-9 and effector Caspase-3, thereby leading to apoptosis [17, 18]. The exogenous apoptosis pathway uses Caspase-8 as the initiator caspase [19]. The apoptosis inhibitor X-linked inhibitor of apoptosis protein (Xiap) binds to Smac to inhibit the initiation phase of apoptosis, and to Caspase-3 to inhibit the execution phase of apoptosis [20]. Forkhead box protein A2 (Foxa2) is involved in the regulation of apoptosis, and previous research has shown that glutathione can mitigate arsenic-induced apoptosis in hepatocytes by activating the Foxa2 pathway [21]. In addition, glutathione depletion can increase the activity of Caspase-3 and Caspase-9 in renal collecting duct cells, leading to apoptosis [22]. The above research showed that glutathione is closely related to apoptosis, which is worthy of further study.

Hypoxic stress leads to a significant accumulation of ROS, resulting in structural damage and dysfunction of the ER [23]. Under stress conditions, unfolded proteins accumulate in the ER, causing ER stress and activating the unfolded protein response (UPR) [24]. The UPR response involves three signaling pathways, namely the protein kinase RNA-like ER kinase (PERK), inositol requiring kinase 1 (IRE1) and activating transcription factor 6 (ATF6) signaling pathways [25]. Related research revealed that hypoxia causes endoplasmic reticulum stress in oligodendrocytes and endothelial cells [26, 27]. HMG-CoA reductase degradation protein 1 (Hrd1) mediates the efflux of misfolded proteins from the endoplasmic reticulum [28], and together with suppressor/Enhancer of lin-12-like (Sel1l) forms the Sel1l-Hrd1 complex, which identifies and degrades misfolded proteins within the ER [29]. A previous study found that hypoxia resulted in elevated Hrd1 protein levels in mouse renal tubular cells [30]. At present, no research has explored the beneficial effects of glutathione against hypoxia-induced ER stress. Glutathione is decomposed into cysteine and glycine in cells. A study has shown that cysteine treatment induced a decrease in the protein level of Atf6 in the brain tissues of mice with hypoxic-ischemic injury [31]. Additionally, glycine treatment was found to alleviate ER stress caused by homocysteine by reducing the protein levels of glucose-regulated protein 78 (GRP78) and C/EBP family homologous protein (CHOP) within endothelial cells [32]. Given these findings, glutathione may mitigate ER stress. Hence, relevant research is warranted.

Grass carp constitute an important component of the fish consumer Market due to their rapid growth, fresh meat, and affordable price. However, its farming process is highly susceptible to hypoxic conditions. Our previous growth experiment indicated that 437.85 mg/kg of glutathione promoted growth in juvenile grass carp. The present study, extends these findings by conducting a hypoxic stress test [3]. This study aimed to investigate the effects of glutathione on gill tissue damage in juvenile grass carp under hypoxic stress and to explore its possible mechanisms of action in terms of tissue oxidative damage, apoptosis, autophagy, and endoplasmic reticulum stress. The optimal levels of glutathione supplementation under hypoxic stress were investigated, providing a reference for the application of glutathione in aquatic feeds.

## Materials and methods

### Ethics statement

All animal procedures were approved by the Animal Care Advisory Committee of Sichuan Agricultural University (China, No. ZSH-2022214018).

### Experimental design and hypoxic stress experiment

**Fig. 1:**
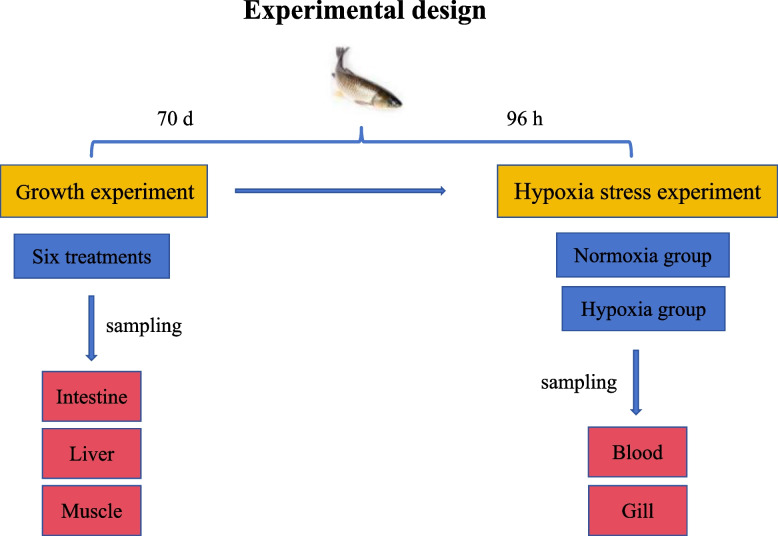
Experimental design diagram

This growth experiment employed the same dietary formulation and fish as in our previous study [3]. The experiment was set up with six treatments, six replicates per treatment, and 60 fish per replicate. The trial demonstrated that glutathione significantly enhanced grass carp growth [3]. The final average weights across the six groups are displayed in Table 1. At the end of the growth test, 60 fish were selected from each treatment and were further divided into the normoxic (30 fish) and hypoxic (30 fish) groups. The experimental conditions (including oxygen concentration and duration) were determined following the methods of Huang et al. [33]. In the normoxic group, an aerator was used for continuous oxygenation to maintain dissolved oxygen above 6 mg/L. For the hypoxic group, a timer-controlled aerator was utilized for intermittent oxygenation, maintaining dissolved oxygen at approximately 1 mg/L. The hypoxic stress experiment was performed for 96 h. The experimental design is shown in Fig. 1.

**Table 1:** Whole-body nutritional components and nutrient conversion rates of juvenile grass carp

Items	Dietary glutathione level, mg/kg					
	0.00	145.95	291.90	437.85	583.80	729.75
FW, g/fish^1^	266.97 ± 1.55^a^	294.47 ± 16.50^b^	322.79 ± 14.43^c^	383.46 ± 3.34^e^	346.95 ± 4.18^d^	321.80 ± 14.06^c^
PER^2^	3.10 ± 0.02^a^	3.17 ± 0.17^ab^	3.26 ± 0.15^bc^	3.33 ± 0.03^c^	3.25 ± 0.04^bc^	3.19 ± 0.14^abc^
Moisture, %	76.19 ± 0.97^c^	73.19 ± 1.70^a^	73.24 ± 1.25^a^	72.68 ± 0.42^a^	74.86 ± 1.31^b^	74.75 ± 0.53^b^
Crude protein, %	13.28 ± 0.53^a^	15.51 ± 0.89^c^	15.59 ± 0.63^c^	15.94 ± 0.42^c^	14.40 ± 0.82^b^	14.48 ± 0.39^b^
Crude lipid, %	7.07 ± 0.47^a^	7.98 ± 0.66^b^	8.09 ± 0.68^b^	8.40 ± 0.45^b^	7.84 ± 0.53^b^	7.08 ± 0.39^a^
Crude Ash, %	3.83 ± 0.10^a^	3.98 ± 0.20^ab^	4.12 ± 0.13^b^	4.16 ± 0.21^b^	4.10 ± 0.19^b^	3.96 ± 0.17^ab^
PRV^2^, %	41.62 ± 2.05^a^	49.80 ± 3.19^c^	52.69 ± 2.46^d^	58.09 ± 1.27^e^	50.25 ± 2.27^cd^	46.33 ± 1.33^b^
LPV^2^, %	148.41 ± 7.49^a^	171.51 ± 16.95^b^	182.28 ± 8.09^b^	204.81 ± 15.79^c^	183.16 ± 9.94^b^	151.33 ± 7.73^a^
ARV^2^, %	44.78 ± 2.40^a^	47.47 ± 2.17^a^	52.48 ± 3.96^b^	56.60 ± 3.82^c^	53.43 ± 2.36^bc^	47.30 ± 2.95^a^

^1 ^FW Final weight. The details regarding fish weight indicators can be found in our previous study [3]

^2^ *PER* Protein efficiency ratio, *PRV* Protein retention value, *LPV* Lipid production value, *ARV* Ash retention value

Data are expressed as mean ± standard deviation (*n* = 6), and different lowercase letters in the same row indicate significant differences (*P* < 0.05)

PER = weight gain (g/fish)/protein intake (g/fish)

PRV (%) = 100 × [final body protein − initial body protein]/protein intake

LPV (%) = 100 × [final body lipid − initial body lipid]/lipid intake

ARV (%) = 100 × [final body ash − initial body ash]/ash intake

### Sample collection and keeping

Upon completion of the growth experiment, the experimental fish were dissected to collect intestinal, liver, and muscle tissue samples, which were then stored at −20 °C for subsequent laboratory analysis. After the hypoxia stress test, the fish were anesthetized, and blood and the gill tissues were promptly collected after dissection. The gill tissue samples included one piece of gill from the same position on the same side of each fish and were stored at −20 °C to determine biochemical indices and −80 °C to determine molecular indices. In addition, 4% paraformaldehyde was used to preserve the samples for histological observation, and 2.5% glutaraldehyde was utilized for the ultrastructural observation of tissue.

### Histological and ultrastructural observations

For histological observation, gill tissues fixed in 4% paraformaldehyde were rinsed under running water to remove residual fixative. Tissue specimens were dehydrated in gradient alcohol and embedded in paraffin wax. Sections were prepared using a microtome, and H&E staining was performed. The structural characteristics of gill tissues were then examined under a Nikon TS100 optical microscope.

In this experimental procedure, a JEM-1400-FLASH transmission electron microscope (TEM) was employed to characterize the tissue ultrastructure. Tissue specimens were removed from 2.5% glutaraldehyde and then fixed with 1% osmium tetroxide. Subsequent processing included dehydration, infiltration, embedding, polymerization, ultrathin sectioning and staining. The prepared samples were finally visualized and captured under the TEM.

### Analysis of blood, whole fish body composition and tissue biochemical parameters

Blood samples treated with sodium heparin anticoagulant were diluted 200-fold with physiological saline to prepare an erythrocyte suspension. An appropriate amount of the cell suspension was then aspirated using a pipette and transferred into the counting chamber of a hemocytometer, after which erythrocytes were counted under an optical microscope. To analyze the osmotic fragility of erythrocytes, different concentrations of sodium chloride solution were prepared (0.28%, 0.32%, 0.36%, 0.40%, 0.44%, and 0.48%). Blood samples pretreated with 1% sodium heparin were added dropwise, gently shaken and mixed, and the concentration of sodium chloride solution at the time of erythrocyte hemolysis was observed and recorded after 2 h.

The proximate composition of diets and whole fish samples was analyzed following the AOAC method [34]. Moisture was determined by the 105 °C drying method, crude protein was determined by the Kjeldahl method, crude fat was determined by the Soxhlet extraction method, and crude ash was determined by 550 °C burning method. The crude protein, crude fat, and crude ash contents of whole fish were determined before and after the growth experiment to calculate the PRV, LPV and ARV.

A 10% tissue homogenate was prepared using 4 °C saline and centrifuged. The supernatant was collected to determine tissue ROS (E004-1-1), malondialdehyde (MDA, A003-1-2), protein carbonyls (PC, A087-1-2) and GSH (A006-2-1)contents, as well as tissue total antioxidant capacity (T-AOC, A015-1-2) and glutamylcysteine ligase (GCL, A120-1-1), superoxide dismutase (SOD, A001-1-2), glutathione peroxidase (GSH-Px, A005-1-2), catalase (CAT, A007-1-1), glutathione sulfotransferase (GST, A004-1-1) and glutathione reductase (GR, A062-1-1) activities. The kits used to determine the above indicators were purchased from Nanjing Jiancheng Bioengineering Institute (Nanjing, China). ROS content was assessed by the chemical fluorescence method. Moreover, the PC and GSH contents, along with the activities of the above-mentioned enzymes, were measured via colorimetric assays; the MDA content was evaluated by the TBA method.

### Real-time quantitative PCR analysis

Using the RNAiso Plus kit, total RNA was extracted from gill tissue samples, and cDNA was produced via reverse transcription with the Prime Script^®^ RT kit. A thermal cycler was used for qPCR analysis, and quantification was carried out using the 2^−ΔΔCT^ method. The primer sequences are listed in Table S1 (Additional file 1), with β-actin as the internal reference.

### Immunofluorescence

Paraffin-embedded tissue samples were taken to prepare white slices (polylysine slides were used to prevent tissue detachment). The sections were dewaxed with xylene solution, rehydrated with gradient alcohol, inactivated with 3% H_2_O_2_ solution, and repaired with heat antigen. The sections were blocked with 5% BSA, followed by incubation with primary antibodies. The primary antibodies used in the experiments included p-IRE1, Hrd1, and Sel1l (1:100 dilution, Abclonal, China). Subsequently, the samples were incubated with fluorescent secondary antibodies, followed by DAPI staining (containing a fluorescence quencher). Finally, an inverted fluorescence microscope (Lecia, DMI4000B, Germany) was used for imaging, and the ImageJ system was used for quantitative analysis.

### Western blot analysis

The protein of gill tissues was extracted by RIPA lysate. After separation by SDS-PAGE, target proteins were transferred to PVDF membranes, blocked using protein-free rapid blocking solution, and then successively incubated with primary and secondary antibodies. The antibodies utilized in this experiment are listed in Table S2 (Additional file 1). Thereafter, the results were visualized using the ECL kit (Oriscience) and imaged with the ChemiDoc™ imaging system. Finally the protein bands were quantitatively analyzed using ImageJ software.

### Statistical analysis

Statistical analyses were conducted using SPSS 27.0 software. Data from the normoxic and hypoxic groups were determined to conform to a normal distribution using a normality test. The data were analyzed using one-way ANOVA and Duncan's multiple comparisons. Independent samples *t*-test was used to compare the normoxic and hypoxic groups, and two-way ANOVA was used to test the main effects of glutathione level, dissolved oxygen, and their interaction effects. The 0.00 mg/kg glutathione group (normoxic and hypoxic group) was set as the negative control group, and the 437.85 mg/kg glutathione group (normoxic and hypoxic group) was set as the positive control group. The results are expressed as mean ± SD, and a *P*-value of less than 0.05 was considered statistically significant. Finally, based on the gill ROS and PC contents, a suitable model was selected for regression analysis to ascertain the optimal addition level of glutathione.

## Results

### Proximate compositions and nutrient deposition of whole fish

The effects of glutathione on whole-body nutritional composition and nutrient deposition rate of juvenile grass carp are presented in Table 1. As glutathione levels increased, whole fish moisture first decreased, reached a minimum at 437.85 mg/kg, and then increased. Moreover, the addition of glutathione (291.90–583.80 mg/kg) significantly increased crude protein, crude fat and crude ash contents of whole fish, and increased the PER, PRV, LPV and ARV of grass carp (*P* < 0.05).

### Tissue redox homeostasis

**Fig. 2:**
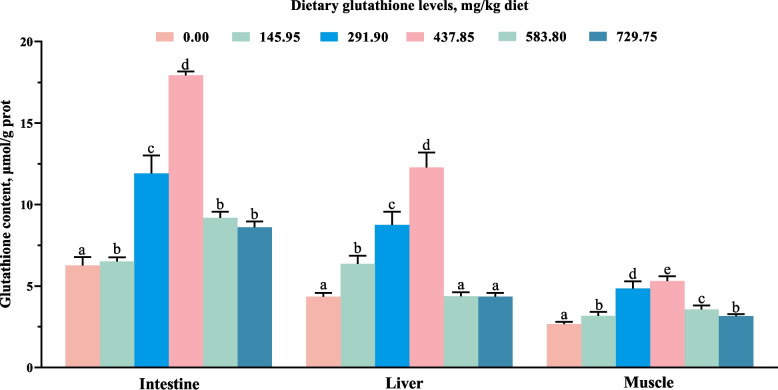
Glutathione content of the intestine, liver and muscle tissues. Different lowercase letters in the bar indicate significant differences (*P* < 0.05)

**Fig. 3:**
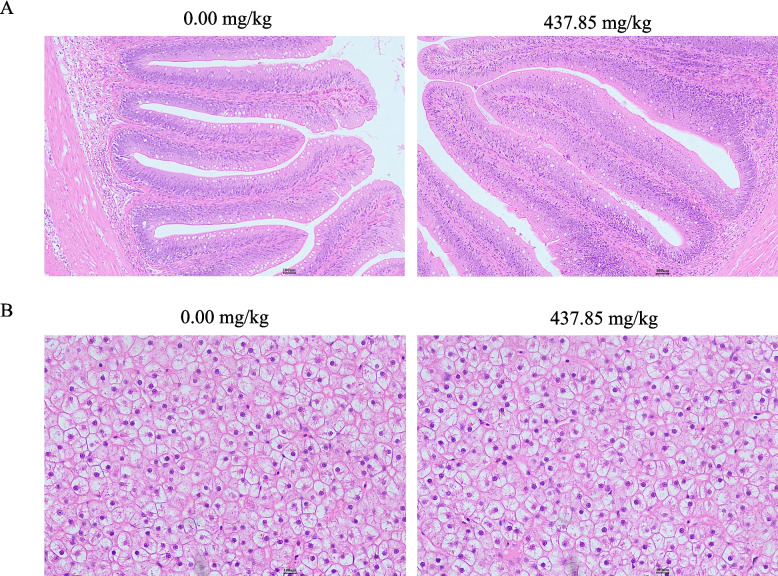
Intestine (**A**) and liver (**B**) H&E staining (100 ×, 400 ×)

Table 2 displays the parameters related to oxidative damage and antioxidant status in intestinal, liver, and muscle tissues. Dietary supplementation with 291.90–437.85 mg/kg glutathione significantly reduced MDA and PC levels, while enhancing the activities of SOD, CAT, GPx, GST, GR, and T-AOC in intestinal, liver and muscle tissues (*P* < 0.05). Figure 2 presents the glutathione content within the three tissues. As the levels of glutathione increased, glutathione content in the three tissues first rose, peaked at 437.85 mg/kg, and then declined. Figure 3 shows the HE staining results of the intestine and liver. No pathological symptoms were found in the intestinal and liver tissues of the 0.00 and 437.85 mg/kg glutathione group, with clear and intact contours of hepatocytes, and structurally intact intestinal tissues.

**Table 2:** The intestine, liver and muscle tissues oxidative damage and antioxidant related parameters of juvenile grass carp

Items	Dietary glutathione level, mg/kg					
	0.00	145.95	291.90	437.85	583.80	729.75
Intestine						
MDA, nmol/mg prot	5.35 ± 0.40^b^	5.07 ± 0.08^ab^	4.92 ± 0.35^a^	5.02 ± 0.19^ab^	5.14 ± 0.34^ab^	5.13 ± 0.07^ab^
PC, nmol/mg prot	10.83 ± 0.62^c^	9.63 ± 0.64^b^	8.31 ± 0.43^a^	8.09 ± 0.63^a^	9.15 ± 0.65^b^	9.40 ± 0.91^b^
T-AOC, mmol/g prot	0.46 ± 0.02^a^	0.53 ± 0.04^b^	0.66 ± 0.04^c^	0.66 ± 0.04^c^	0.55 ± 0.05^b^	0.51 ± 0.03^b^
SOD, U/mg prot	66.62 ± 3.92^a^	67.11 ± 7.34^a^	68.10 ± 6.60^a^	76.67 ± 1.20^b^	72.68 ± 7.07^ab^	70.55 ± 4.91^ab^
CAT, U/mg prot	2.42 ± 0.09^a^	2.64 ± 0.10^b^	3.58 ± 0.12^e^	3.69 ± 0.14^e^	3.30 ± 0.09^d^	3.12 ± 0.08^c^
GPx,U/mg prot	235.38 ± 20.16^a^	248.75 ± 11.92^a^	309.73 ± 29.98^b^	377.67 ± 33.12^c^	257.56 ± 25.90^a^	263.12 ± 17.14^a^
GST, U/mg prot	44.02 ± 3.32^a^	47.62 ± 4.38^ab^	55.85 ± 4.59^c^	51.37 ± 4.92^bc^	48.45 ± 2.86^ab^	44.94 ± 3.21^a^
GR, U/g prot	0.98 ± 0.07^a^	1.19 ± 0.06^b^	1.90 ± 0.14^d^	2.29 ± 0.16^e^	1.73 ± 0.13^c^	1.14 ± 0.08^b^
Liver						
MDA, nmol/mg prot	6.68 ± 0.76^e^	6.39 ± 0.53^e^	5.73 ± 0.25^d^	1.73 ± 0.13^b^	0.87 ± 0.06^a^	3.29 ± 0.18^c^
PC, nmol/mg prot	8.63 ± 0.62^d^	7.22 ± 0.49^c^	6.30 ± 0.61^b^	5.50 ± 0.34^a^	6.42 ± 0.16^b^	6.63 ± 0.38^b^
T-AOC, mmol/g prot	1.39 ± 0.03^ab^	1.45 ± 0.04^b^	1.46 ± 0.06^b^	1.41 ± 0.03^ab^	1.36 ± 0.05^a^	1.35 ± 0.07^a^
SOD, U/mg prot	46.84 ± 3.94^a^	47.55 ± 4.29^a^	48.12 ± 4.80^a^	50.18 ± 3.95^a^	47.88 ± 4.60^a^	46.80 ± 4.48^a^
CAT, U/mg prot	12.43 ± 0.04^a^	12.87 ± 0.04^d^	13.87 ± 0.05^e^	12.70 ± 0.04^c^	12.51 ± 0.04^b^	12.41 ± 0.06^a^
GPx,U/mg prot	564.12 ± 29.97^a^	609.96 ± 43.55^b^	663.98 ± 8.56^c^	622.55 ± 17.22^b^	619.32 ± 60.92^b^	617.76 ± 16.92^b^
GST, U/mg prot	12.38 ± 1.23^a^	14.87 ± 0.84^b^	17.23 ± 1.54^c^	19.87 ± 1.24^d^	16.66 ± 0.59^c^	14.85 ± 1.08^b^
GR, U/g prot	1.13 ± 0.06^ab^	1.18 ± 0.05^b^	1.29 ± 0.06^c^	1.33 ± 0.06^c^	1.19 ± 0.11^b^	1.09 ± 0.08^a^
Muscle						
MDA, nmol/mg prot	3.53 ± 0.35^c^	3.30 ± 0.37^c^	2.67 ± 0.14^b^	2.28 ± 0.14^a^	2.76 ± 0.17^b^	2.74 ± 0.29^b^
PC, nmol/mg prot	5.89 ± 0.55^b^	5.88 ± 0.50^b^	4.22 ± 0.19^a^	3.84 ± 0.24^a^	4.27 ± 0.20^a^	4.30 ± 0.30^a^
T-AOC, mmol/g prot	1.07 ± 0.04^a^	1.08 ± 0.03^ab^	1.13 ± 0.05^bc^	1.13 ± 0.03^bc^	1.15 ± 0.06^c^	1.08 ± 0.04^ab^
SOD, U/mg prot	30.83 ± 1.52^a^	38.10 ± 3.35^d^	34.06 ± 3.33^bc^	36.21 ± 2.18^cd^	35.32 ± 2.84^bcd^	32.37 ± 1.36^ab^
CAT, U/mg prot	0.40 ± 0.03^a^	0.51 ± 0.07^b^	0.53 ± 0.06^b^	0.73 ± 0.06^c^	0.52 ± 0.05^b^	0.50 ± 0.05^b^
GPx,U/mg prot	425.83 ± 16.16^a^	471.06 ± 27.93^b^	582.46 ± 15.11^d^	533.15 ± 16.27^c^	535.63 ± 24.67^c^	492.16 ± 4.02^b^
GST, U/mg prot	5.48 ± 0.35^a^	11.45 ± 0.98^b^	16.52 ± 0.45^de^	17.21 ± 0.41^e^	16.09 ± 0.97^d^	14.14 ± 1.22^c^
GR, U/g prot	0.31 ± 0.02^a^	0.47 ± 0.02^c^	0.53 ± 0.02^d^	0.67 ± 0.03^e^	0.53 ± 0.02^d^	0.35 ± 0.03^b^

Data are expressed as mean ± standard deviation (*n* = 6), and different lowercase letters in the same row indicate significant differences (*P* < 0.05)

### Blood biochemical parameters

The results of the RBC count and osmotic fragility are presented in Table 3. Glutathione supplementation (291.90–437.85 mg/kg) significantly increased erythrocyte counts and decreased the osmotic fragility of erythrocytes (first and complete hemolysis) in the normoxic and hypoxic groups (*P* < 0.05). The results from the two-way ANOVA indicated that both glutathione concentrations and dissolved oxygen, as well as their interactions, significantly influenced the RBC count and osmotic fragility (complete hemolysis *P* < 0.05).

**Table 3:** Blood biochemical parameters

Items		Dietary glutathione level, mg/kg						*P-*value (Two way ANOVA)		
		0.00	145.95	292.90	437.85	583.80	729.75	DO	GSH	DO × GSH
RBC count	N	1.57 ± 0.14^a^	1.63 ± 0.13^ab^	1.84 ± 0.13^bc^	1.88 ± 0.23^c^	1.77 ± 0.27^abc^	1.67 ± 0.09^abc^	< 0.01	< 0.01	< 0.05
	H	1.56 ± 0.08^a^	1.85 ± 0.24^b^	2.25 ± 0.20^c*^	2.41 ± 0.33^c*^	1.93 ± 0.27^b^	1.89 ± 0.24^b^			
Osmotic fragility at first hemolysis	N	0.40 ± 0.00^ab^	0.37 ± 0.02^ab^	0.37 ± 0.02^ab^	0.36 ± 0.04^a^	0.39 ± 0.02^ab^	0.42 ± 0.03^b^	< 0.01	< 0.01	0.87
	H	0.43 ± 0.03^b^	0.40 ± 0.00^ab^	0.39 ± 0.02^a^	0.40 ± 0.00^ab^	0.40 ± 0.00^ab^	0.43 ± 0.03^b^			
Osmotic fragility in complete hemolysis	N	0.35 ± 0.02^b^	0.28 ± 0.00^a^	0.31 ± 0.02^ab^	0.29 ± 0.02^a^	0.31 ± 0.02^ab^	0.31 ± 0.02^ab^	< 0.01	< 0.01	< 0.05
	H	0.35 ± 0.02^bc^	0.31 ± 0.02^ab^	0.31 ± 0.02^ab^	0.29 ± 0.02^a^	0.35 ± 0.02^bc^	0.39 ± 0.02^c*^			

*RBC count* Red blood cell count (10^12^/L). Osmotic fragility at first hemolysis: sodium chloride solution %; osmotic fragility in complete hemolysis: sodium chloride solution %. *N* Denotes normoxia group, *H* Denotes hypoxia group. Data are expressed as mean ± standard deviation (*n* = 6), with different lowercase letters in the normoxia and hypoxia groups representing significant differences (*P* < 0.05), and * indicating significant differences between the normoxia and hypoxia groups at the same glutathione level (*P* < 0.05)

### Glutathione metabolism and transport in gill tissue

**Fig. 4:**
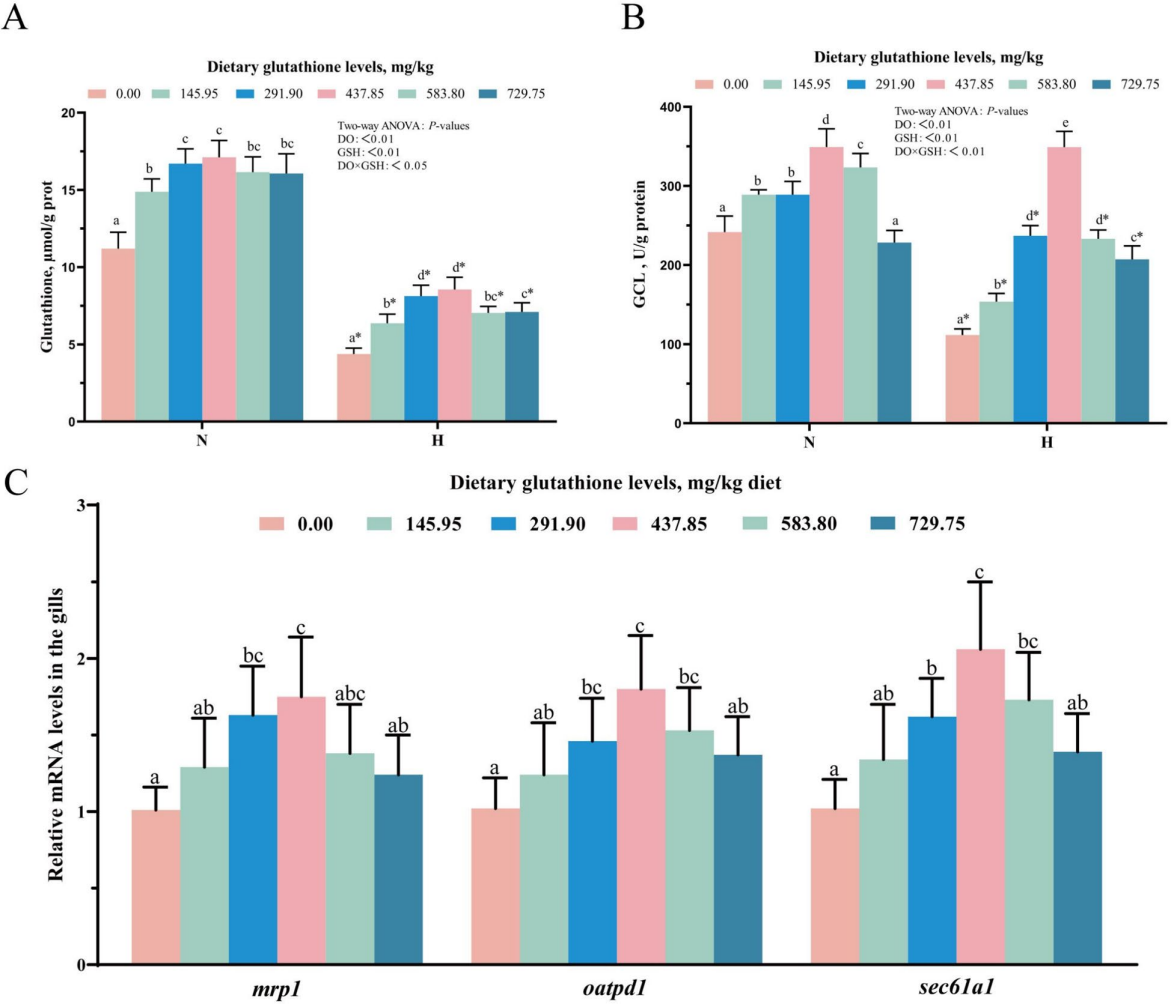
Effects of glutathione levels on GSH content (**A**), GCL activity (**B**) and glutathione transporter mRNA levels (**C**) in gill tissue. N indicates normoxia group and H indicates hypoxia group. Data were expressed as mean ± standard deviation (*n* = 6), different lowercase letters in the normoxia and hypoxia groups represent significant differences (*P* < 0.05), and for the same glutathione level, * denotes a significant difference between the normoxia and hypoxia groups (*P* < 0.05)

Figure 4 reflects the impacts of glutathione levels on glutathione metabolism and transport in gill tissue. Dietary supplementation with 291.90–583.80 mg/kg glutathione significantly increased gill tissue GSH content and GCL activity in normoxic and hypoxic groups. The independent samples t-test indicated that at equivalent glutathione levels, GSH content and GCL activity were significantly reduced in the hypoxic group compared to the normoxic group (*P* < 0.05). Two-way ANOVA results revealed that the glutathione level, dissolved oxygen level, and the interaction effect of the two exerted a significant effect on GSH content and GCL activity in gills (*P* < 0.05). Finally, the levels of mRNA *mrp1* and *oatpd1* (trans-plasma membrane transporters) along with *sec61a1* (trans-endoplasmic reticulum membrane transporter), in gill tissue first rose and then declined as the glutathione levels increased, peaking at 437.85 mg/kg.

### Gill tissue structure

**Fig. 5:**
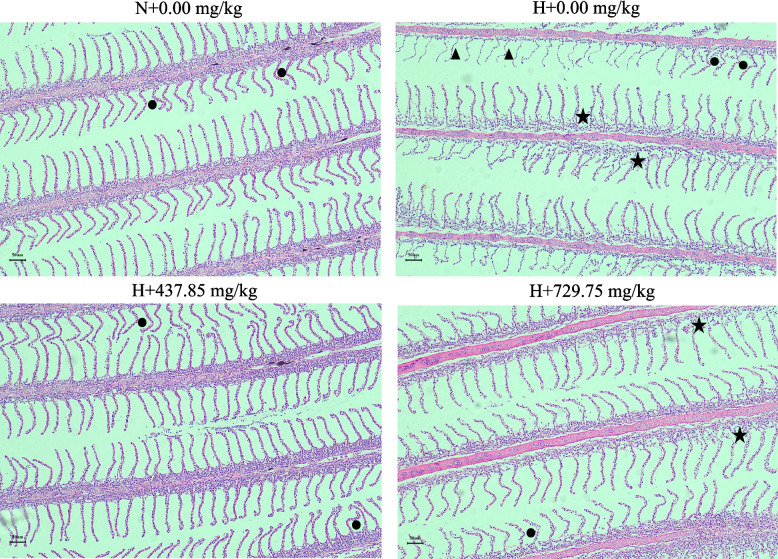
Gill H&E staining (100 ×). ★: gill epithelial cells floating away; ▲: gill epithelial cells necrotic; ●: gill lamellae bent. N indicates normoxia group, H indicates hypoxia group

**Fig. 6:**
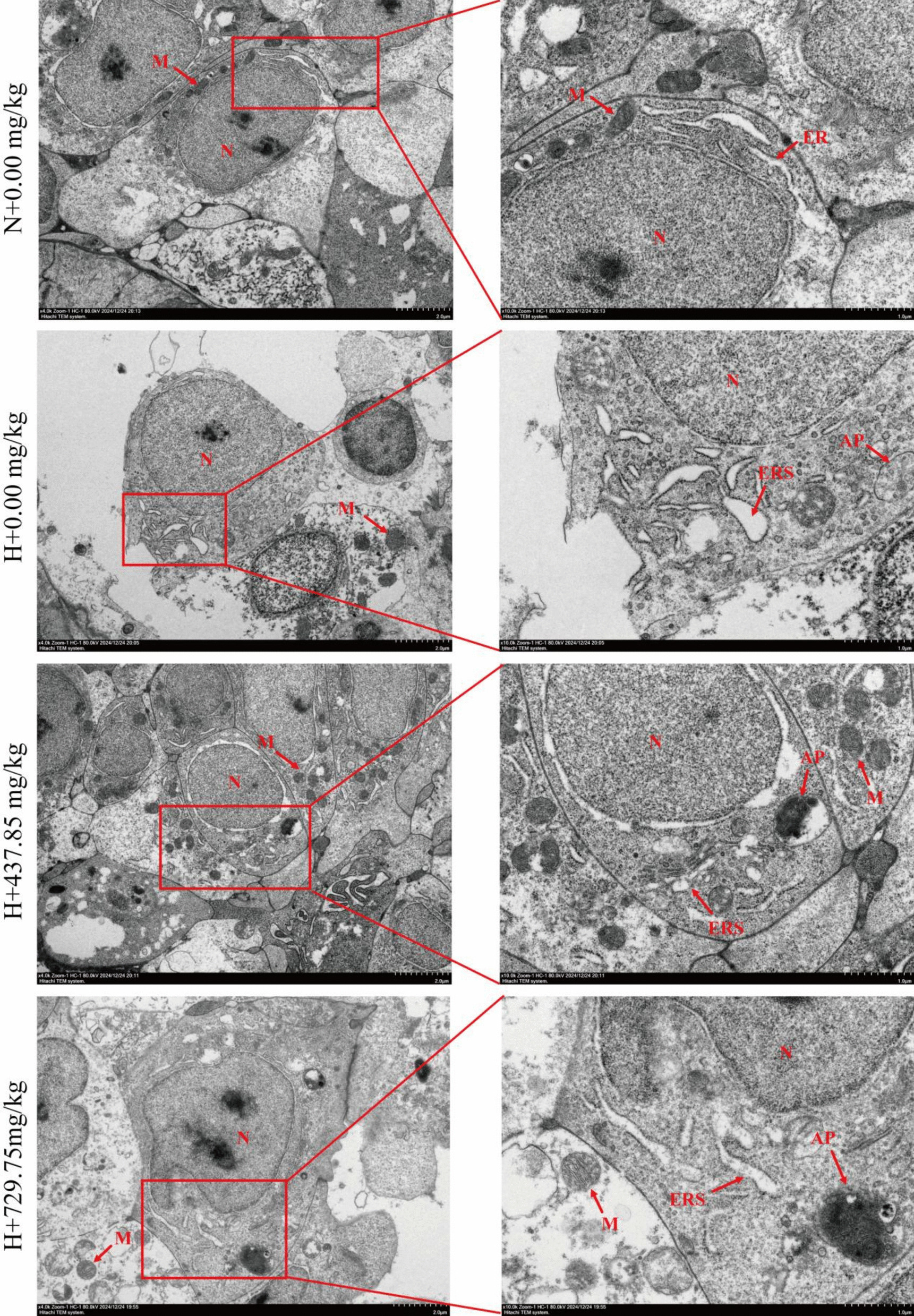
Transmission electron micrographs of gill cells (4,000 ×, 10,000 ×). N: Nucleus; M: Mitochondrion; ER: Endoplasmic reticulum; ERS: Endoplasmic reticulum swelling AP: Autophagosome. N indicates normoxia group, H indicates hypoxia group

Figure 5 illustrates the results of H&E staining of gill tissue. In the normoxic group, the gill tissue showed slight bending of gill lamellae, showing gill epithelial cells with normal morphology, intact structure, and tight adherence to the gill lamellae. After hypoxic stress, the gill tissues of the negative control group showed severe pathological changes, including detachment and floating of gill epithelial cells, necrosis, fusion of secondary gill flaps, and bending of gill lamellae. In contrast, supplementation with 437.85 mg/kg glutathione alleviated these symptoms. Secondly, Fig. 6 displays the transmission electron microscopy results of gill cells. Compared with the normoxic + 0.00 mg/kg glutathione group, the gill tissue structure of the hypoxic + 0.00 mg/kg glutathione group was severely damaged, and obvious swelling of the endoplasmic reticulum and a small number of autophagosomes were observed, whereas supplementation with 437.85 mg/kg glutathione alleviated the above symptoms.

### Biochemical indicators of gill tissue

**Fig. 7:**
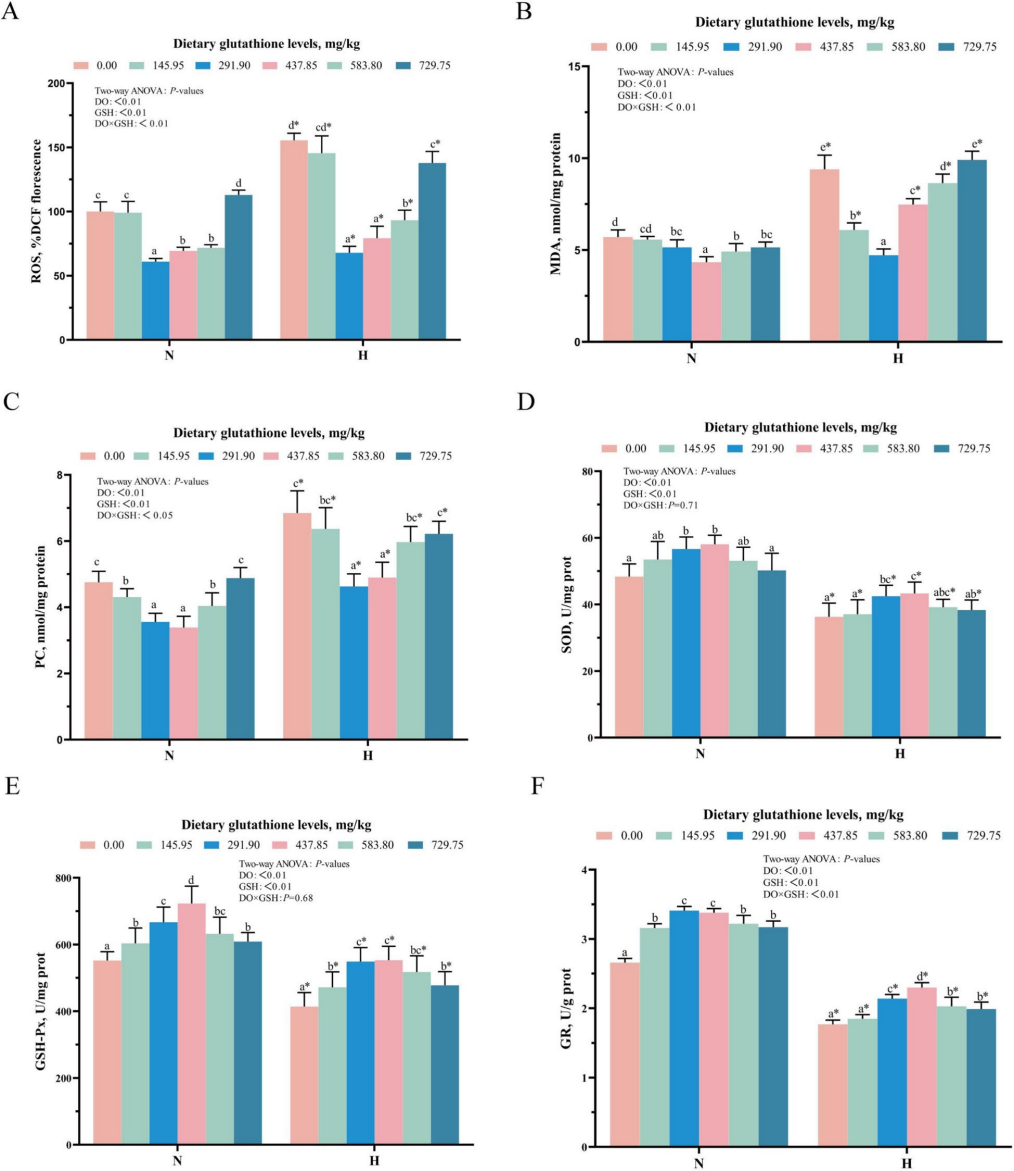
Effect of glutathione levels on parameters related to oxidative damage and antioxidant in gill. **A** ROS: reactive oxygen species (%DCF fluorescence intensity); **B** MDA: malondialdehyde (nmol/mg prot); **C** PC: protein carbonyl (nmol/mg prot); **D** SOD: superoxide dismutase (U/mg prot); **E** GSH-Px: glutathione peroxidase (U/mg prot); **F** GR: glutathione reductase (U/g prot). Data were expressed as mean ± standard deviation (*n* = 6), different lowercase letters in the normoxia and hypoxia groups represent significant differences (*P* < 0.05), and for the same glutathione level, * denotes a significant difference between the normoxia and hypoxia groups (*P* < 0.05)

Figure 7 shows the results of oxidative damage and antioxidant-related indexes in gill tissues. As the dietary glutathione levels rose, ROS, MDA, and PC contents initially declined, reaching their lowest at 437.85 or 291.90 mg/kg, and then began to rise. Conversely, SOD, GSH-Px, and GR activities first increased, achieving a peak at 437.85 mg/kg, and then declined. Compared with the normoxic group, the hypoxic group showed a remarkable rise in gill ROS, MDA, and PC levels, whereas SOD, GSH-Px and GR activities were notably decreased (*P* < 0.05). Additionally, the interaction between glutathione and dissolved oxygen levels exerted a significant impact on ROS, MDA, and PC contents as well as GR activity (*P* < 0.05).

### Apoptosis in gills

**Fig. 8:**
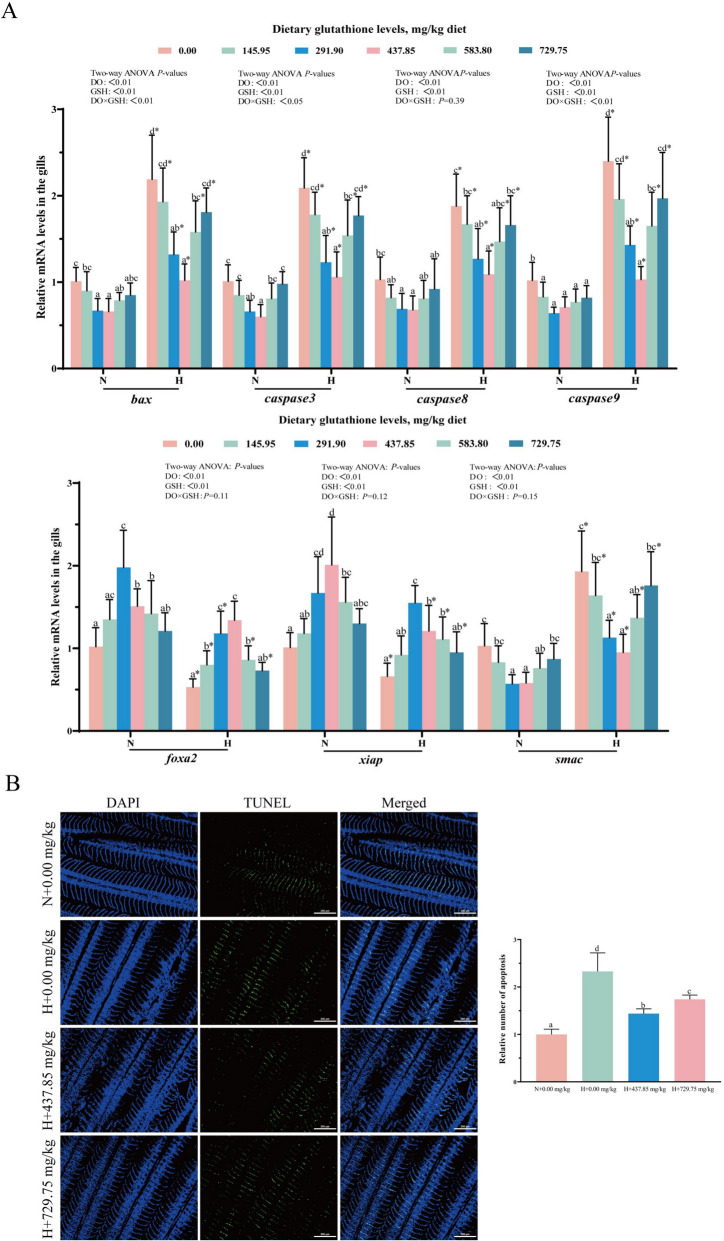
Effect of glutathione on apoptosis-related indexes in gill tissue. **A** mRNA levels of apoptosis-related genes; **B** TUNEL staining. Blue fluorescence indicates nuclei, green fluorescence indicates apoptotic cells. Data were expressed as mean ± standard deviation (*n* = 6), different lowercase letters in the normoxia and hypoxia groups represent significant differences (*P* < 0.05), and for the same glutathione level, * denotes a significant difference between the normoxia and hypoxia groups (*P* < 0.05)

The results of apoptosis-related gene expression levels in gill tissue are displayed in Fig. 8A. Supplementation with 437.85 mg/kg glutathione significantly reduced gill *bax*, *caspase3*, *caspase8*, *caspase9*, and *smac* mRNA levels, and elevated *foxa2* and *xiap* mRNA levels (*P* < 0.05). At equal glutathione levels, the mRNA levels of *smac*, *bax*, *caspase3*, *caspase8* and *caspase9* were markedly higher in the hypoxia group than in the normoxic group (*P* < 0.05). Furthermore, the interplay of glutathione and dissolved oxygen levels had a notable effect on *bax*, *caspase3* and *caspase9* mRNA levels (*P* < 0.05). TUNEL staining of gill tissues is shown in Fig. 8B. The number of apoptotic cells was significantly higher in the hypoxic + 0.00 mg/kg glutathione group compared to the normoxic + 0.00 mg/kg glutathione group, while supplementation with 437.85 mg/kg glutathione remarkably reduced their number (*P* < 0.05).

### Autophagy in gills

**Fig. 9:**
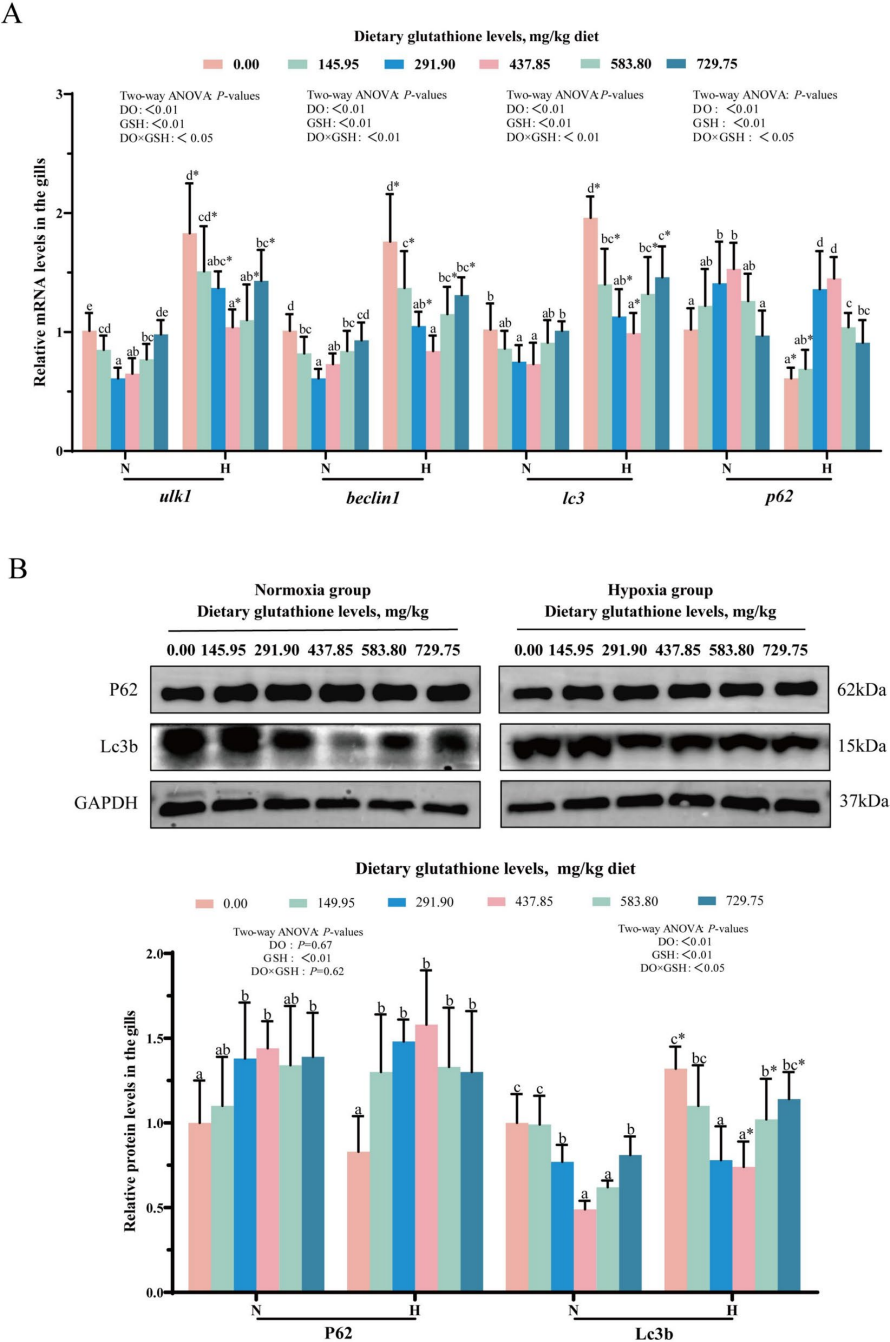
Effects of glutathione levels on mRNA levels (**A**) and protein levels (**B**) of autophagy-related genes in gill tissue cells. Data were expressed as mean ± standard deviation (*n* = 6), different lowercase letters in the normoxia and hypoxia groups represent significant differences (*P* < 0.05), and for the same glutathione level, * denotes a significant difference between the normoxia and hypoxia groups (*P* < 0.05)

The results of gill tissue autophagy-related gene mRNA levels are shown in Fig. 9A. Supplementation with 291.90–437.85 mg/kg glutathione markedly reduced *ulk1*, *beclin1* and *lc3* mRNA levels, while elevating *p62* mRNA levels in gill tissues (*P* < 0.05). At the same level of glutathione supplementation, *ulk1*, *beclin1,* and *lc3* mRNA levels were notably higher in the hypoxic group compared with the normoxic group (*P* < 0.05). Additionally, glutathione, dissolved oxygen concentrations, and their interactive effect exerted significant impacts on *ulk1*, *beclin1* and *lc3* and *p62* mRNA levels (*P* < 0.05). The results of autophagy-related protein levels in gill tissue are displayed in Fig. 9B. Specifically 437.85 mg/kg glutathione significantly raised P62 protein levels and reduced Lc3b protein levels (*P* < 0.05). Finally, Lc3b protein levels were markedly impacted by the interplay of glutathione and dissolved oxygen levels (*P* < 0.05).

### Endoplasmic reticulum stress in gills

**Fig. 10:**
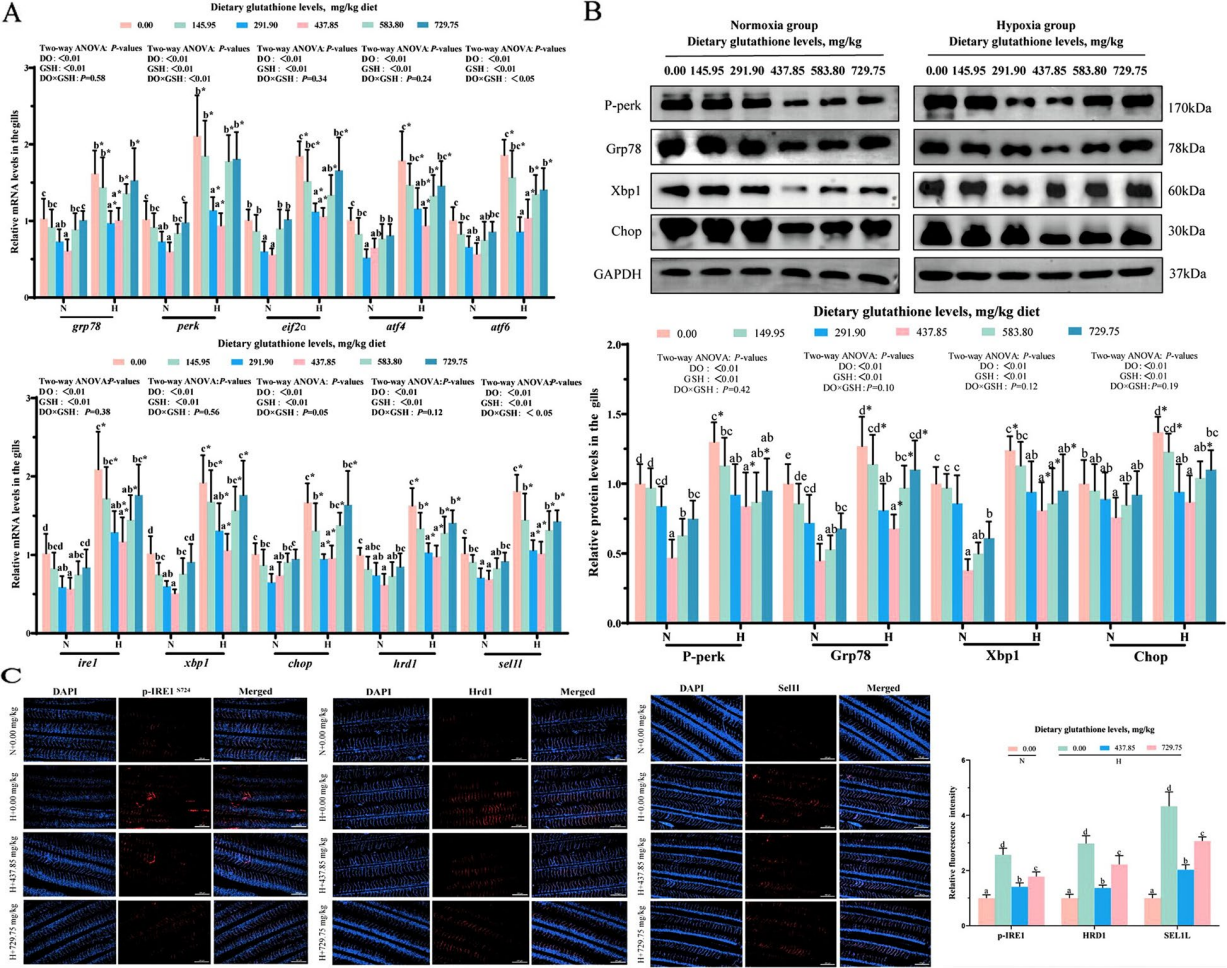
Effect of glutathione level on endoplasmic reticulum stress in gill tissue. **A** mRNA levels of endoplasmic reticulum stress-related genes; **B** Levels of endoplasmic reticulum stress-related proteins; **C** Immunofluorescence plots and their quantitative results, blue fluorescence indicates DAPI nuclear staining, and red fluorescence represents p-IRE1, Hrd1, and Sel1l proteins, respectively. Data were expressed as mean ± standard deviation (*n* = 6), different lowercase letters in the normoxia and hypoxia groups represent significant differences (*P* < 0.05), and for the same glutathione level, * denotes a significant difference between the normoxia and hypoxia groups (*P* < 0.05)

The effects of glutathione levels on gill endoplasmic reticulum stress are demonstrated in Fig. 10. Supplementation with 437.85 mg/kg glutathione markedly reduced *grp78*, *perk*, *eif2ɑ*, *atf4*, *ire1*, *xbp1*, *atf6*, *chop*, *hrd1* and *sel1l* mRNA levels along with P-perk, Grp78, Xbp1 and Chop protein levels in gill tissue (*P* < 0.05). The mRNA levels of the aforementioned genes were significantly higher in the hypoxic group compared to the normoxic group under the same level of glutathione (*P* < 0.05). The results of two-way ANOVA revealed that *perk*, *atf6*, *chop,* and *sel1l* mRNA levels were markedly influenced by glutathione, dissolved oxygen levels and their interaction (*P* < 0.05). Finally, immunofluorescence analysis (Fig. 10C) revealed that the fluorescence intensities of p-IRE1, Hrd1, and Sel1l proteins were significantly enhanced in the hypoxic group compared to the normoxic group (*P* < 0.05). In contrast, supplementation with 437.85 mg/kg glutathione remarkably reduced the fluorescence intensity of these proteins in the hypoxic group (*P* < 0.05).

### Correlation and regression analysis

Table 4 presents the correlation analysis of the relevant indicators. Within the hypoxia group, *perk*, *ire1* and *atf6* were positively correlated with the mRNA levels of *grp78*, while P-perk and Xbp1 were positively correlated with the protein levels of Grp78 (*P* < 0.01). Moreover, *beclin1* was positively correlated with the mRNA levels of *ulk1* and *lc3* (*P* < 0.01).

**Table 4:** Correlation analysis of endoplasmic reticulum stress and autophagy-related indexes in gill tissue of grass carp under hypoxia stress

Independent variable	Implicit variable	Correlation coefficient	Significance (*P*)
*grp78* mRNA level	*perk* mRNA level	0.740	< 0.001
	*ire1* mRNA level	0.630	< 0.001
	*atf6* mRNA level	0.721	< 0.001
Grp78 protein level	P-perk protein level	0.719	< 0.001
	Xbp1 protein level	0.694	< 0.001
*ulk1* mRNA level	*beclin1* mRNA level	0.747	< 0.001
*beclin1* mRNA level	*lc3* mRNA level	0.682	< 0.001
Lc3b protein level	*beclin1* mRNA level	0.503	= 0.002

**Fig. 11:**
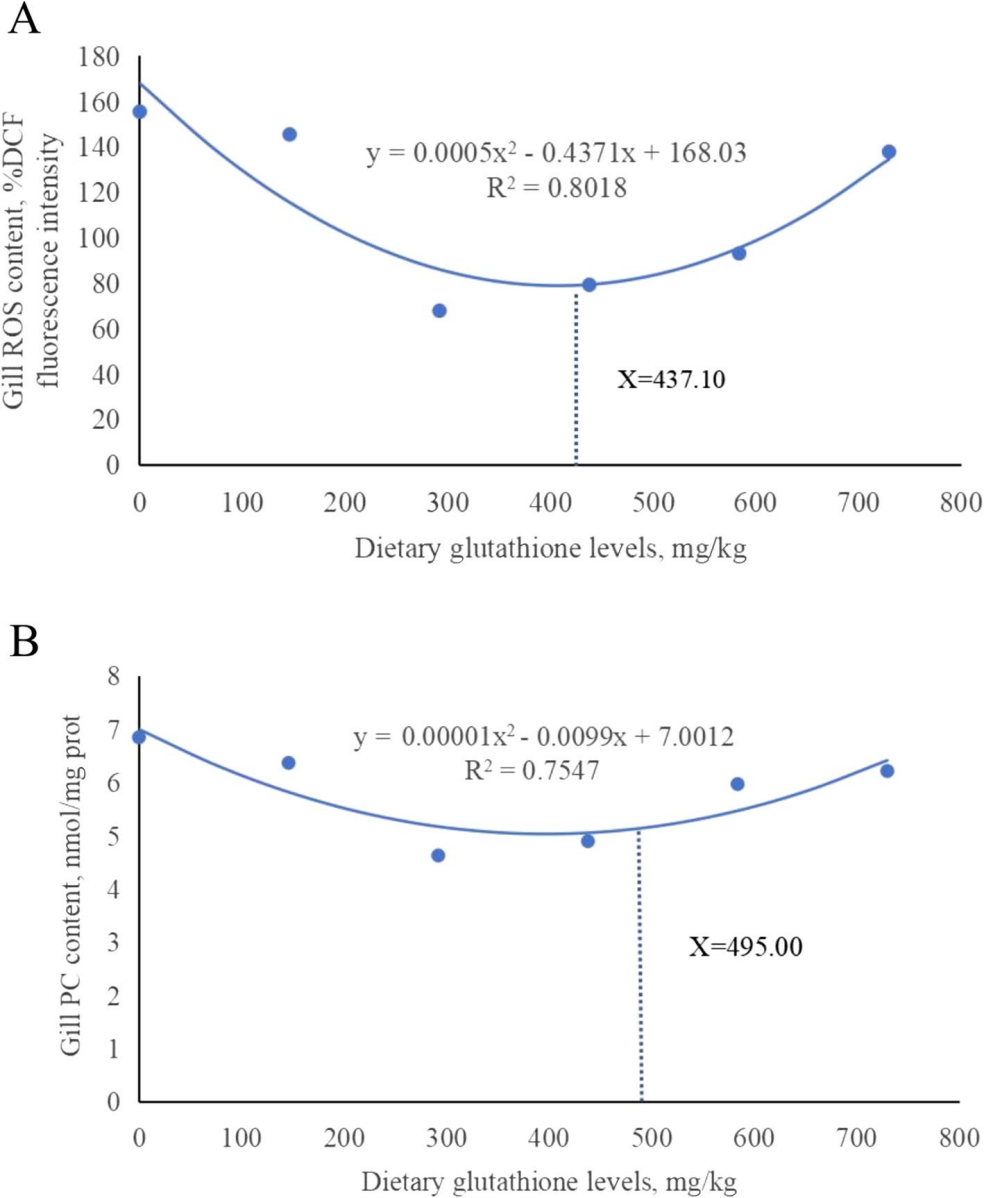
Quadratic regression analysis between glutathione levels and ROS and PC contents in gill tissue of juvenile grass carp. **A** ROS content; **B** PC content

Using gill tissue ROS and PC levels as markers, quadratic regression analysis (Fig. 11) determined the optimal dietary glutathione supplementation levels for mitigating hypoxic stress in juvenile grass carp at 437.10 and 495.00 mg/kg, respectively.

## Discussion

### Glutathione improved the production performance and antioxidant capacity of fish

Fish growth depends on nutrient deposition [35]. Our findings revealed that the addition of glutathione elevated protein retention value and lipid production value, suggesting that glutathione could enhance nutrient deposition in grass carp. Antioxidant enzymes play an essential role in the maintenance of redox homeostasis [36]. This research demonstrated that dietary supplementation with 291.90–437.85 mg/kg glutathione effectively lowered MDA and PC levels in intestinal, liver, and muscle tissues while boosting antioxidant enzyme activities. Collectively these findings imply an improved antioxidant defense system in grass carp, which is consistent with earlier observations in *Litopenaeus vannamei* [37].

### Glutathione improved glutathione metabolism and transport in gill tissue

Glutathione is synthesized in cells catalyzed by GCL and glutathione synthase (GSS), with glutamylcysteine ligase being the rate-limiting enzyme for this reaction. In this experiment, GSH content and GCL activity in gills were reduced after hypoxia stress, suggesting that hypoxia May inhibit glutathione biosynthesis, whereas supplementation with 291.90 to 583.80 mg/kg glutathione elevated GSH levels and GCL activity, thereby alleviating hypoxia-induced glutathione depletion. Studies have shown that glutathione transport across the plasma membrane is dependent on multidrug resistance protein 1 (Mrp1) and organic anion transporting polypeptides 1 (Oatp1) [38, 39]. A previous study reported that the Sec61 protein on the endoplasmic reticulum membrane acts as a channel for glutathione to enter the endoplasmic reticulum [40]. In this research, supplementation with 291.90 to 437.85 mg/kg glutathione elevated the mRNA levels of gill glutathione transporter proteins (*mrp1*, *oatpd1* and *sec61a1*). This suggests that appropriate levels of glutathione promote the ability of glutathione to enter the gills.

### Glutathione attenuated gill tissue damage under hypoxia stress

Gills are in direct contact with the aquatic environment and are susceptible to damage from hypoxic stress. A study on silver carp (*Hypophthalmichthys molitrix*) revealed that hypoxic stress resulted in gill lamella bending and gill filament splitting [41]. In this research, hypoxic stress led to gill epithelial cell detachment and necrosis, which were attenuated by glutathione. Excessive ROS attacked intracellular lipids and proteins, resulting in the formation of MDA and PC, inducing oxidative stress and leading to tissue damage [42, 43]. In the present study, hypoxic stress resulted in elevated levels of ROS, MDA, and PC in gills, while supplementation with 291.90 to 437.85 mg/kg glutathione reduced their contents, indicating that glutathione alleviated oxidative damage in gill tissue. Moreover, hypoxic stress resulted in reduced activities of SOD, GSH-Px and GR in gills, whereas supplementation with 437.85 mg/kg glutathione elevated their activities, suggesting that glutathione enhanced the antioxidant activity of gills. In summary, glutathione may reduce gill tissue injury by attenuating hypoxic stress-induced oxidative damage as well as increasing antioxidant capacity.

### Glutathione attenuated gill apoptosis under hypoxia stress

Caspases are pivotal regulators of apoptosis, with Caspase-8 and Caspase-9 belonging to the initiator caspases and Caspase-3 to the effector caspases [44]. The pro-apoptotic protein Bax activates the mitochondrial pathway of apoptosis by inducing mitochondrial outer membrane permeabilization [45]. In our research, hypoxic stress increased the number of apoptotic cells and elevated *caspase3*, *caspase8*, *caspase9*, and *bax* mRNA levels in gills, whereas supplementation with glutathione reduced the number of apoptotic cells and decreased *caspase3*, *caspase8*, *caspase9*, and *bax* mRNA levels. These results suggested that glutathione attenuated hypoxia stress-induced apoptosis. Glutathione was found to attenuate lipopolysaccharide-induced hepatopancreatic apoptosis in a study of the *Eriocheir sinensis* [46], which was consistent with our findings. Xiap combines with Caspase-3 and inhibits its activity, thereby inhibiting apoptosis [47]. Smac is a pro-apoptotic agent that promotes apoptosis by combining with Xiap and disrupting Xiap's inhibition of Caspase-3 [48]. This research found that hypoxia stress reduced *xiap* mRNA levels while simultaneously increasing *smac* mRNA levels in gill tissue. Conversely, when 291.90 to 583.80 mg/kg glutathione was added, *xiap* mRNA levels rose and *smac* mRNA levels declined, indicating that glutathione inhibited gill apoptosis.

### Glutathione attenuated gill autophagy under hypoxia stress

Autophagy is a tightly regulated cellular process that degrades and recycles dysfunctional organelles and other cellular materials through the lysosomal pathway [49]. The initiation and nucleation of autophagy are regulated by Ulk1 and Beclin1. Notably, Ulk1 could phosphorylate Beclin1 and promote autophagy [50, 51]. In this research, *ulk1* and *beclin1* mRNA levels were elevated after hypoxic stress. Conversely, supplementation with appropriate levels of glutathione resulted in, a decrease in their mRNA levels. The extended phase of autophagy is controlled by the LC3 coupling system [52]. The results of this experiment showed that hypoxic stress resulted in elevated *lc3* mRNA levels and Lc3b protein levels. Meanwhile, appropriate levels of glutathione reduced their mRNA and protein levels. During the degradation phase, p62 binds to LC3 and promotes aggregation of autophagic substrates, while p62 itself is degraded by autophagosomes [53]. This research demonstrated that hypoxic stress resulted in a reduction in the levels of *p62* mRNA and protein within the gills, while supplementation with glutathione elevated its mRNA and protein levels. In conclusion, hypoxic stress induced autophagy in gills, whereas glutathione attenuated autophagy.

### Glutathione alleviates gill endoplasmic reticulum stress under hypoxic stress

The endoplasmic reticulum is a key organelle for protein folding processing and quality control, and its homeostasis is critical for normal cellular physiological function. Stress stimulation causes the accumulation of unfolded proteins in the ER, which triggers ER stress and activates the UPR response. CHOP is an endoplasmic reticulum stress-specific transcription factor, which can be activated by ATF4, XBP1 and ATF6 under ER stress [54]. The results of this research demonstrated that *atf4*, *atf6*, *xbp1* and *chop* mRNA levels as well as Xbp1 and Chop protein levels were elevated following hypoxic stress. However, supplementation with 437.85 mg/kg glutathione significantly reduced their mRNA and protein levels. GRP78, a chaperone protein found in the endoplasmic reticulum, serves as a hallmark indicator of UPR response activation [55]. Under ER stress, PERK, IRE1, and ATF6 dissociate from GRP78, which in turn activates the UPR response [56]. Our research found that *grp78*, *perk* and *ire1* mRNA levels, as well as P-perk and Grp78 protein levels, were elevated after hypoxic stress, whereas the addition of 291.90–437.85 mg/kg reduced their mRNA and protein levels. Therefore, glutathione appears to mitigate ER stress caused by hypoxia by inhibiting the PERK, IRE1, and ATF6 pathways.

### Appropriate addition of glutathione

The quadratic regression analysis of ROS and PC levels in gill tissue indicated that the optimal levels of dietary glutathione additions were 437.10 and 495.00 mg/kg, respectively. However, the optimal glutathione addition level identified by PC exceeded that of ROS. This discrepancy may be attributed to ROS being directly scavenged by glutathione; hence, the increase in ROS level leads to increased protein carbonylation level, showing that cells are subjected to more serious oxidative damage. In order to repair or protect damaged proteins, cells need higher levels of glutathione.

## Conclusion

In summary, the present study found that 291.90–437.85 mg/kg glutathione effectively improves production performance and antioxidant capacity in fish, and attenuates hypoxic stress-induced gill tissue damage. The findings suggest that: (1) Glutathione may alleviate gill damage caused by hypoxia by inhibiting tissue oxidative damage, apoptosis, autophagy, and ER stress. (2) The optimum supplementation of glutathione was 437.10 and 495.00 mg/kg, based on ROS and PC levels in gill tissue as markers, respectively. Our results provide insights into the possible mechanisms by which glutathione alleviates hypoxic stress. Glutathione could be used as a potential feed additive to mitigate the adverse effects of hypoxia on aquaculture.

## Supplementary Information

Additional file 1: Table S1 Real-time PCR primer sequences. Table S2 Antibodies information.

## Data Availability

The datasets are included in this article and available from the corresponding author on reasonable request.

## Abbreviations

Atf4: Activating transcription factor 4
Atf6: Activating transcription factor 6
Bax: BC12 associated X protein
Beclin1: Coiled-coil myosin-like BCL2-interacting protein
Chop: C/EBP family homologous protein
eIF2ɑ: Eukaryotic translation initiation factor-2α
ERAD: Endoplasmic reticulum associated degradation
Foxa2: Forkhead box protein A2
Grp78: Glucose regulated protein 78
Hrd1: HMG-CoA reductase degradation protein 1
Ire1: Inositol requiring kinase 1
Lc3: Microtubule-associated protein 1 light chain 3
Mrp1: Multidrug resistance protein 1
Oatp1: Organic anion transporting polypeptides 1
p62: Autophagy receptor p62
Perk: Protein kinase RNA-like ER kinase
Sec61a1: Sec61 translocon subunit alpha 1
Sel1l: Suppressor/Enhancer of lin-12-like
Smac: Second mitochondria-derived activator of caspases
Ulk1: UNC-51-like kinase 1
UPR: Unfolded protein response
Xbp1: X-Box binding protein 1
Xiap: X linked inhibitor of apoptosis protein
