# Supplementary material for: Glutathione mitigates hypoxia-induced gill damage in juvenile grass carp (Ctenopharyngodon idellus) by alleviating endoplasmic reticulum stress and autophagy
Source: J Anim Sci Biotechnol. 2025 Nov 6;16:144. doi: 10.1186/s40104-025-01274-x (PMC12590615; doi:10.1186/s40104-025-01274-x)
Supplement: Supplementary file 1 — Additional file 1: Table S1 Real-time PCR primer sequences. Table S2 Antibodies information. [file 40104_2025_1274_MOESM1_ESM.docx]

Table S1 Real-time PCR primer sequences

| Gene | Primer sequence forward | Primer Sequence reverse | Accession number |
| --- | --- | --- | --- |
| β-actin | CGTGACATCAAGGAGAAG | GAGTTGAAGGTGGTCTCAT | M25013 |
| MRP1 | TCTGATCCGTTCTGGGATTGG | CAGGGTATCCAAACCAGCAGT | XM_051888049 |
| Oatp1d1 | TGTCATGCCTCTTGGCTTGT | CCGTCTGTATGAGAGCCAGG | NM_001348086 |
| Sec61a1 | GTCACGAGTGCTGTTTTGGG | TAGAAACGCTTTGCCACTCCT | XM_051898264 |
| Bax | CATCTATGAGCGGGTTCGTC | TTTATGGCTGGGGTCACACA | JQ793788 |
| caspase3 | GCTGTGCTTCATTTGTTTG | TCTGAGATGTTATGGCTGTC | JQ793789 |
| caspase8 | ATCTGGTTGAAATCCGTGAA | TCCATCTGATGCCCATACAC | KM016991 |
| caspase9 | CTGTGGCGGAGGTGAGAA | GTGCTGGAGGACATGGGAAT | JQ793787 |
| Foxa2 | TACGGAGAGCCCGAGTGTTA | TGTTCATGGAGTTCATTCCCAGT | XM_051869066 |
| XIAP | TGGAGCCCATTCAAGGTCAA | CTGTAGGACTTTCTGTGCTGC | [XM_051918473](https://www.ncbi.nlm.nih.gov/entrez/viewer.fcgi?db=nucleotide&id=2326912737) |
| Smac | GCCAACACGTACCTCTCTCA | TGTATGTAGTGCCTTGGCGT | NM_200346 |
| ULK1 | AATTATGATGCCAAAGCTGATC | GGTGTCTTAGGTGACTGGAGGT | AL929056 |
| Beclin1 | CAAACAAGATGGGACTATGC | CAGACCACCTGAGCAATACA | MG821470 |
| LC3 | ATGCCTTCGGAAAAGACATTTAAAC | TTACTGAGGACACGCAGTTCC | MG821471 |
| p62 | TGATGGGGTTGGCTCTTGTGAA | CCTGGAGGGCTAAAGTGGGATG | MK370058 |
| GRP78 | GTCACCTTTGAGATCGACGTG | AGAGAGTAGGCGTAGCTC | FJ436356 |
| PERK | CAGCGTTTACCTGGGGATGT | TTACTGCCCCAGGCGTTTAG | KX906957 |
| eIF2α | ATCAATAGCGGAGATGGGCG | TGATGACCACCACGCATTCA | KJ126860 |
| ATF4 | TTCGGCCAACACCTTAGACC | CTTGCCTCATCTTTCGGGGT | AY437846 |
| IRE1 | GAACGCCACATACTCTGA | TGTCCACTGTCACCACTA | MG797683 |
| XBP1 | TTCTGAGTCCGCAGCAGGTG | GTTCTGGGTCAAGGATGTCC | KU509247 |
| ATF6 | CACCTCTGTTCCTGACCTGA | TAGACAGACAGTGGAGAGGG | KT279356 |
| CHOP | ATCAGAACGAGCGCCTCAAA | TTCACCTCCTGGTGTTGACG | KX013389 |
| Hrd1 | TACCCGACTGTGGTGTACCT | AGGGGAGAAATCGTCCCTGA | XM_051860103 |
| Sel1L | CAGACTGCACTCGGTTTCCT | TCCACCCAAAGCTCCAAAGG | XM_051874559 |

Table S2. Target proteins, dilution factor, antibody cat. no. and antibody source of proteins selected for analyzing by western blotting.

| Target proteins | Dilution ratio | Antibody cat. no. | Antibody source |
| --- | --- | --- | --- |
| GRP78 | 1:2000 | AF5366 | Affinity |
| p-PERK | 1:1000 | AP1501 | ABclonal |
| XBP1 | 1:2000 | A1731 |  |
| CHOP | 1:1000 | A11346 |  |
| LC3B | 1:2000 | ET-170165 | Huabio |
| P62 | 1:6000 | HA721171 |  |
| GAPDH | 1:5000 | ET1601-4 |  |
| Secondary antibody (HRP-conjugated Goat anti-Rabbit IgG) | 1:8000 | AS014 | ABclonal |
